# Account of an Unusual Strangulation of a Portion of the Large Intestines, Caused by the Formation of an Adventitious Band, between the Stomach and Liver

**Published:** 1843-12-09

**Authors:** P. Le B. Stickney

**Affiliations:** Philadelphia


### ACCOUNT OF AN UNUSUAL STRANGULATION OF A PORTION OF THE LARGE INTESTINES,

*Caused by the formation of an adventitious band, between the stomach and liver.*

BY P. LE B. STICKNEY, M. D.

On the 7th instant I was called to attend Mrs. A. in her confinement; she had an easy labour, and was delivered of a dead infant. On the second day after the delivery, the patient was very comfortable, the bowels having been spontaneously and very freely moved. On the third day there were two copious evacuations. Under these circumstances it was not considered necessary to administer the usual laxative on such occasions. On the fourth there was but one evacuation. On the fifth and sixth, there were none. The patient continuing to improve.

On the 14th inst., I was sent for in haste, and found the patient complaining of a severe pain in the back, accompanied with a violent hiccough and vomiting, the pulse natural and skin moist and cool. An ounce of castor oil was ordered, together with injections of warm water and laudanum. The oil was rejected from the stomach, but the pain subsided after using the injections. No operation of the bowels.

On the 15th, the pain had returned, the vomiting and hiccough was constant. The usual remedies were employed, but with little success. The bowels still constipated. Seidlitz powders with mustard cataplasms over the stomach ordered.

On the 16th, the bowels in the same condition; the pulse beating over a hundred; the abdomen greatly distended, and very tender upon pressure. Dr. E. Ward was called in consultation. The patient was bled freely, and a purge of calomel and jalap directed, the action of which was to be hastened by an enema. During the day, as no effect had followed the purgative, one ounce of castor oil and three drachms of the spirits of turpentine were given. As no effect followed the first dose, a like quantity was given about four hours afterwards. In the evening, as the bowels had not yet been moved, a drop of croton oil was given, warm fomentations applied to the abdomen, and the injections repeated.

On the 17th, as the patient was no better, a large catheter was introduced through the rectum, in order if possible to fill the colon with an injection. The resistance to its introduction beyond a certain point, was so great, that the attempt was abandoned.

On the 18th, Dr. J. Pancoast was called in consultation. It was thought proper to attempt giving another injection. Dr. Pancoast introduced the rectum catheter, as far as the sigmoid flexure of the colon, but beyond this there was an obstruction to its passage, which it did not appear prudent to force, and a large quantity of a saline injection was thrown up, but which returned quickly by the side of the catheter, without affording any relief. Examination through the abdominal walls gave no particular indication of disease, except some peritoneal soreness, at the lower part of the cavity. An aromatic infusion of senna was directed, and if this was rejected by the stomach, the patient was to be placed fully under the influence of opium by enema.

On the 19th, no alteration for the better. Dr. Pancoast visited the patient for the second time, in company with Dr. Dunglison, to determine as to the propriety of attempting to relieve the patient, by making a temporary opening into the colon at the right or left lumbar region.

After a careful examination it was decided that the case did not warrant the operation. A suppository of five grains of opium was ordered, this to be followed at repeated intervals by a pill of two grains of opium by the mouth.

On the morning of the 20th the patient died. Twelve hours after death, my friend Dr. C. Huston and myself, made a careful post mortem examination. Upon cutting into the abdomen we found a circumscribed cavity extending from above the umbilicus to the pubis, and bounded in front throughout its whole extent by the parietes of the abdomen. The walls composing the cavity were thickened, and of a chocolate colour. The cavity contained a large quantity of fœtid gas, and about six ounces of a dark coloured fluid. On farther dissection, the whole abdominal cavity being laid open, the transverse portion of the colon was found doubled upon itself, and pushed downwards towards the right crest of the ileum, so as to lie obliquely across the small intestines. On examining the descending colon, a portion of it together with the whole of the sigmoid flexure, was found encircled by a ligamentous band of about an inch in length, and of the size of a crow quill; one extremity being attached to the anterior surface of the stomach, and the other extremity to the apex of the left lobe of the liver, forming a perfect bridle by which this portion of the colon was suspended, and causing a complete strangulation. The upper portion of this strangulated loop being the sigmoid flexure, and lying with its long diameter parallel with the median line of the body, formed the cavity which was first opened in making the dissection.

About one-third of the omentum covered the loop and was adherent to it, and to the walls of the abdomen.

There was also a slight adhesion between the band and the intestines. Had it been possible to have ascertained the state of the parts beforehand, the case might possibly have warranted an incision through the walls of the abdomen for the purpose of dividing the stricture.

*Philadelphia, Dec.* 1843.

